# Non-invasive brain stimulation modulates neural correlates of performance monitoring in patients with obsessive-compulsive disorder

**DOI:** 10.1016/j.nicl.2022.103113

**Published:** 2022-07-14

**Authors:** Luisa Balzus, Julia Klawohn, Björn Elsner, Sein Schmidt, Stephan A. Brandt, Norbert Kathmann

**Affiliations:** a Humboldt-Universität zu Berlin, Department of Psychology, Berlin, Germany; b Humboldt-Universität zu Berlin, Berlin School of Mind and Brain, Berlin, Germany; c Charité – Universitätsmedizin Berlin, Department of Neurology, Berlin, Germany

**Keywords:** OCD, Error monitoring, Error-related negativity, Non-invasive brain stimulation, Transcranial direct current stimulation, Presupplementary motor area

## Abstract

Overactive performance monitoring, as reflected by enhanced neural responses to errors (the error-related negativity, ERN), is considered a biomarker for obsessive-compulsive disorder (OCD) and may be a promising target for novel treatment approaches. Prior research suggests that non-invasive brain stimulation with transcranial direct current stimulation (tDCS) may reduce the ERN in healthy individuals, yet no study has investigated its efficacy in attenuating the ERN in OCD. In this preregistered, randomized, sham-controlled, crossover study, we investigated effects of tDCS on performance monitoring in patients with OCD (*n* = 28) and healthy individuals (*n* = 28). Cathodal and sham tDCS was applied over the presupplementary motor area (pre-SMA) in two sessions, each followed by electroencephalogram recording during a flanker task. Cathodal tDCS reduced the ERN amplitude compared to sham tDCS, albeit this effect was only marginally significant (*p* = .052; mean difference: 0.86 μV). Additionally, cathodal tDCS reduced the correct-response negativity and increased the error positivity. These neural modulations were not accompanied by behavioral changes. Moreover, we found no evidence that the tDCS effect was more pronounced in the patient group. In summary, our findings indicate that tDCS over the pre-SMA modulates neural correlates of performance monitoring across groups. Therefore, this study represents a valuable starting point for future research to determine whether repeated tDCS application induces a more pronounced ERN attenuation and normalizes aberrant performance monitoring in the long term, thereby potentially alleviating obsessive-compulsive symptoms and providing a psychophysiological intervention strategy for individuals who do not benefit sufficiently from existing interventions.

## Highlights

• Effects of tDCS on performance monitoring examined in OCD and healthy individuals.
• A preregistered, randomized, sham-controlled tDCS–EEG study was conducted.
• Cathodal tDCS over the pre-SMA reduced the error-related negativity (ERN).
• Correct-response negativity was enhanced, error positivity reduced by cathodal tDCS.
• The findings substantiate the role of the ERN as a target for new interventions.

## 1 Introduction

Obsessive-compulsive disorder (OCD) is a debilitating psychiatric disorder estimated to affect 2–3% of the population (Ruscio et al., 2010). The disorder is characterized by recurrent intrusive thoughts (obsessions) and repetitive behaviors (compulsions). Core symptoms of OCD, such as doubt whether actions were completed correctly, worry about possible mistakes, and repetitive behaviors, have long been conceptualized as manifestations of an overactive error signaling system (Pitman, 1987). Supporting this notion, research using event-related potentials (ERPs) has consistently shown overactive error monitoring in OCD, as indicated by increased amplitudes of the error-related negativity (ERN; for a meta-analysis, see Riesel, 2019).

The ERN is a negative deflection in the ERP at fronto-central electrode sites that occurs within 100 ms after commission of an error (Gehring et al., 1993). A major neural generator is presumed to be located in the anterior cingulate cortex (Debener et al., 2005), but source loci have also been reported to lie in the presupplementary motor area (pre-SMA; Fu et al., 2019, Grützmann et al., 2016) and the posterior midcingulate cortex (Agam et al., 2011, Buzzell et al., 2017). In terms of functional significance, the ERN is viewed as an alarm signal generated by the performance monitoring system that signals the need for behavioral adjustment to prevent future errors (Ullsperger et al., 2014). The amplitude of the ERN is influenced by motivational factors and individual characteristics, such that it is larger when errors are motivationally salient and in individuals with high levels of trait anxiety or anxiety disorders (for reviews, see Meyer and Hajcak, 2019, Olvet and Hajcak, 2008, Weinberg et al., 2012).

Besides being robustly associated with OCD and anxiety disorders (for reviews and meta-analysis, see e.g., Gillan et al., 2017, Moser et al., 2016, Riesel, 2019, Weinberg et al., 2015), enhanced ERN magnitude is heritable (Anokhin et al., 2008, Riesel et al., 2019b), evident in unaffected first-degree relatives of individuals with OCD or anxiety disorders (Carrasco et al., 2013, Riesel et al., 2011, Riesel et al., 2019b), and insensitive to treatment-induced symptom reduction (Hajcak et al., 2008, Kujawa et al., 2016, Ladouceur et al., 2018, Riesel et al., 2015a). Therefore, ERN enhancement is considered a biomarker for OCD and anxiety disorders (Gillan et al., 2017, Riesel, 2019). Moreover, the ERN has been shown to be predictive of later development of anxiety disorders (Meyer et al., 2015, Meyer et al., 2018), suggesting associations with psychological risk mechanisms that render individuals more sensitive to psychosocial stressors and increase risk for psychopathology (Banica et al., 2021, Riesel et al., 2021). Thus, considering its direct relation to a probable risk mechanism in the pathogenesis of OCD and anxiety disorders, the ERN may be a suitable target for novel intervention and prevention approaches (Meyer et al., 2020). Even though cognitive behavioral therapy and pharmacological approaches are highly effective treatments for OCD, about 50% of patients do not benefit sufficiently from existing interventions (Kathmann et al., 2022, Öst et al., 2015), underscoring the need for additional treatment strategies. Evidence indicates that standard treatment approaches, such as cognitive behavioral therapy, do not affect the ERN and performance-related worry (e.g., Ladouceur et al., 2018, Riesel et al., 2015a), suggesting that the increase in ERN amplitude and possibly the heightened perceived aversiveness of errors persist. Therefore, standard treatment approaches could be complemented by novel intervention strategies that directly target the ERN and may thereby reduce obsessive-compulsive or anxiety symptoms and/or the risk of developing such psychopathology (Hajcak et al., 2019, Klawohn et al., 2020a, Meyer, 2022). Even beyond putative effects on symptoms of psychopathology, modulation of aberrant error monitoring is informative for a better understanding of pathomechanisms and the identification of potential targets for psychophysiological intervention strategies. Therefore, we aimed to examine whether error monitoring can be modulated in patients with OCD by non-invasive brain stimulation.

Previous research has shown that the ERN can be modulated in individuals with OCD by experimental manipulations, at least on a short-term basis. Specifically, limiting cognitive resources by dual-task demands and reallocating attention by cognitive training procedures such as attentional bias modification have been shown to temporarily attenuate the ERN in adults (Klawohn et al., 2016, Klawohn et al., 2020a) and adolescents (Tan et al., 2021) with OCD, whereas symptom provocation and social responsibility contexts have been found to increase the ERN in patients with OCD (Roh et al., 2017) and individuals with OCD symptoms (Jansen & de Bruijn, 2020). In contrast, other experimental manipulations, such as monetary punishment of errors (Endrass et al., 2010) or task instructions emphasizing accuracy over speed (Riesel et al., 2019a), have failed to effectively modulate the ERN in patients with OCD, suggesting that in OCD, adaptability of error-related neural activity to situational demands is limited. Thus, strategies to effectively and sustainably modulate aberrant error monitoring in clinical populations still need to be determined.

Techniques of non-invasive brain stimulation, such as transcranial direct current stimulation (tDCS), may be a promising approach to rebalance abnormal activation patterns and normalize overactive error signals in OCD in the long term. TDCS modulates cortical excitability of a targeted area via application of a low-intensity direct current. Effects of tDCS are polarity-dependent, with anodal tDCS being generally thought to increase and cathodal tDCS to decrease cortical excitability, by depolarizing or hyperpolarizing the resting membrane potential, respectively (Nitsche & Paulus, 2000). Prior research suggests that tDCS targeting the pre-SMA modulates the ERN in healthy individuals within a single session, such that anodal tDCS increases, whereas cathodal tDCS decreases the ERN (Reinhart and Woodman, 2014, Verveer et al., 2021; but see Bellaïche et al., 2013). In line with this, ERN attenuation has also been observed after inhibitory pre-SMA stimulation by low-frequency repetitive transcranial magnetic stimulation (rTMS; Rollnik et al., 2004). Regarding clinical populations, tDCS has been found to normalize the reduced ERN in patients with schizophrenia (Reinhart et al., 2015). Moreover, it has been reported that deep brain stimulation of the anterior limb of the internal capsule and nucleus accumbens attenuates the ERN in patients with OCD (Sildatke et al., 2022). No study has yet investigated the effects of tDCS on error monitoring in OCD.

Notably, a separate line of research suggests that tDCS has the potential to reduce OCD symptoms in patients who do not respond to conventional treatments. Inhibitory protocols involving cathodal tDCS over the pre-SMA appear particularly promising in terms of therapeutic efficacy (for reviews, see e.g., Brunelin et al., 2018, Rapinesi et al., 2019). For a successful use and improvement of therapeutic efficacy, it is essential to elucidate the underlying mechanism by which tDCS may alleviate obsessive-compulsive symptoms. Therefore, this study aimed to investigate whether error monitoring can be modulated by tDCS in patients with OCD and healthy individuals. To this end, cathodal and sham tDCS was applied over the pre-SMA in two separate sessions, each followed by electroencephalogram (EEG) recording during performance of a flanker task.

Our hypotheses were based on reported effects of cathodal tDCS on performance monitoring in healthy individuals (Reinhart & Woodman, 2014). Our primary hypothesis was that compared to sham tDCS, cathodal tDCS would reduce the ERN amplitude across healthy individuals and patients with OCD. In addition, we expected that if inhibitory pre-SMA stimulation by cathodal tDCS attenuates performance monitoring processes, error rates would be increased and behavioral adaptation after error commission, that is, post-error slowing (PES), would be reduced. Moreover, based on previous findings (Reinhart & Woodman, 2014), we predicted that cathodal tDCS would increase the amplitude of the error positivity (Pe), a centro-parietal positivity that follows the ERN and has been related to error awareness and motivational error significance (Falkenstein et al., 2000, Overbeek et al., 2005). To explore effects of cathodal tDCS on performance monitoring of correct responses, we additionally analyzed the correct-response negativity (CRN; Ford, 1999). The CRN is a negative deflection after correct responses that is similar to the ERN but smaller, and has been reported to be increased in OCD as well, although less consistently (for a review, see Michael et al., 2021).

Regarding group differences, we predicted increased ERN amplitudes in patients with OCD compared to healthy participants in the sham condition. In line with previous experimental modulations of the ERN in OCD (Klawohn et al., 2016, Klawohn et al., 2020a), we expected the tDCS-induced ERN attenuation to be more pronounced in the patient group compared to the control group. Hypotheses, experimental design, sample size, and analysis plan of this study were preregistered on the Open Science Framework (https://osf.io/7z8hj/), in line with recent efforts in ERP research to promote Open Science practices (Clayson et al., 2019, Clayson et al., 2022).

## 2 Methods

### 2.1 Participants

The sample size was determined based on a priori power analyses. We estimated the sample size required to allow replication of the findings reported by Reinhart and Woodman (2014). Based on an effect size of Cohen’s *d_z_* = 0.60 (for behavioral performance) and *d_z_* = 0.91 (for ERN amplitude), and a significance level of 5% (two-sided), a sample of 24 participants per group provides 80% power to detect an effect of tDCS in both groups.

In a simulation-based power analysis with 1000 simulations using the SIMR package in R (Version 1.0.5; Green & MacLeod, 2016), we additionally estimated whether for a sample of 24 participants per group, there would be sufficient power to detect a tDCS effect on ERN amplitude in a linear mixed model analysis, which is the analysis method used in this study. An ERN reduction of 1.5 μV, which is at the lower end of previously reported ERN modulations (Klawohn et al., 2016, Klawohn et al., 2020a, Reinhart and Woodman, 2014, Roh et al., 2017), would be detected with a power of 95.40% (95% confidence interval [CI]: 93.91, 96.61).

Since estimating the sample size based on effect sizes reported in a previous study often leads to underpowered studies (Anderson et al., 2017), and to ensure sufficient power in case of dropouts and data loss due to poor data quality, we adopted a conservative approach and recruited a larger sample of 30 participants per group.

The recruited sample comprised 30 patients with OCD and 30 healthy control participants. Patients with OCD and control participants were individually matched for gender, age, and level of education (see Table 1). Five control participants and one patient dropped out after the first session (control participants without giving a reason; patient chose to discontinue due to discomfort following the first session), and were replaced according to our preregistered recruitment strategy. Two patients were retrospectively identified to meet one of the exclusion criteria specified below (*n* = 1 comorbid bipolar disorder; *n* = 1 presence of orthodontic retainer), and were excluded pairwise with their matched controls. No participant had to be excluded due to poor EEG data quality (i.e., excessive alpha activity or > 25% of segments discarded as artifact) or an insufficient number of incorrect responses (i.e., < 6) to reliably quantify the ERN (Olvet & Hajcak, 2009). The final sample consisted of 28 patients with OCD and 28 healthy control participants.

**Table 1:** Demographic and Clinical Characteristics of Patients With Obsessive-Compulsive Disorder (OCD) and Healthy Control (HC) Participants.

Characteristic	Patients with OCD (*n* = 28)	HC participants (*n* = 28)	Test statistic a	*p*
Age (years)	33.29 (8.57)	33.07 (8.20)	*t*(53.90) = −0.10	.924
Gender (*n* female:male)	17:11	17:11	χ^2^(1) = 0.00	1.000
Years of education b	12.14 (1.46)	12.14 (1.08)	*t*(49.74) = 0.00	1.000
BDI-II	14.14 (11.34)	1.86 (2.69)	*t*(30.03) = −5.58	< .001
OCI-R	25.75 (9.95)	6.25 (5.65)	*t*(42.75) = −9.02	< .001
Y-BOCS total score	23.36 (3.84)	–	–	–
Y-BOCS obsessions	11.43 (1.81)	–	–	–
Y-BOCS compulsions	11.86 (2.53)	–	–	–

*Note.* Values are means with standard deviations in parentheses except for gender. BDI-II = Beck Depression Inventory-II; OCI-R = Obsessive-Compulsive Inventory-Revised; Y-BOCS = Yale-Brown Obsessive Compulsive Scale.

a *t* refers to Welch’s *t* test.

b Years of education refer to primary and secondary education, not to higher education.

Patients were recruited from the specialized OCD outpatient clinic at Humboldt-Universität zu Berlin, where they were currently waiting for (*n* = 23) or undergoing cognitive behavioral therapy (*n* = 5; number of sessions completed at assessment *M* = 26.20, *SD* = 11.26, range = 9–40). All patients were diagnosed with OCD as the primary diagnosis, as assessed with the Structured Clinical Interview for DSM-IV (SCID-I, German version; Wittchen et al., 1997) by trained clinical psychologists. Twenty patients had at least one comorbid psychiatric disorder, including major depressive disorder (*n* = 3 current episode; *n* = 10 remitted), dysthymia (*n* = 3), social phobia (*n* = 4), specific phobia (*n* = 2), panic disorder (*n* = 1), generalized anxiety disorder (*n* = 1), and bulimia nervosa (*n* = 1). Thirteen patients were currently receiving psychotropic medication (*n* = 10, selective serotonin reuptake inhibitors [SSRIs]; *n* = 1, serotonin–norepinephrine reuptake inhibitor; *n* = 2, combination of SSRIs and other antidepressants).

Healthy control participants were recruited through public advertisement. General inclusion criteria for all participants were age between 18 and 65 years and normal or corrected-to-normal vision. Exclusion criteria for all participants were: lifetime diagnosis of any psychotic, bipolar, or substance-related disorder; use of benzodiazepines in the last week or of neuroleptic medication in the last three months; history of head trauma or neurological disease; any general exclusion criteria for tDCS, such as any metal or electronic implants in the head or upper body, skin disorder or skin condition at or near stimulation locations, or current pregnancy. Further exclusion criteria for healthy control participants were any current or past psychiatric disorder (assessed with a SCID-I screening questionnaire) or current or past psychotherapeutic treatment. All participants provided written informed consent prior to participation. Participants received monetary compensation or course credit for participation. Study procedures were in accordance with the ethical guidelines of the Declaration of Helsinki and approved by the local ethics committee at Humboldt-Universität zu Berlin (protocol number 2019-02). This study was registered in the German Clinical Trials Register (ID: DRKS00016807).

### 2.2 Procedure

The study employed a randomized, double-blind, sham-controlled, crossover design. Participants were invited for two experimental sessions and received one session of cathodal and one of sham tDCS. The order of tDCS conditions was randomized and counterbalanced across participants. Immediately after the stimulation, the EEG cap was mounted and participants performed a flanker task while EEG was recorded. The time interval between stimulation and the flanker task was approximately 15 min (*M* = 13:34 min, *SD* = 3:05, range = 7:55–24:45). Sessions were conducted at least 5 days apart (*M* = 7.25 days, *SD* = 1.25, range = 5–12). Participants and experimenters were blind to the tDCS condition during both sessions.

At the beginning of the first session, severity of obsessive-compulsive symptoms was assessed in all patients using the German version of the Yale-Brown Obsessive Compulsive Scale (Y-BOCS; Goodman et al., 1989, Hand and Büttner-Westphal, 1991). All participants completed the Obsessive-Compulsive Inventory-Revised (OCI-R; Foa et al., 2002, Gönner et al., 2008) and the Beck Depression Inventory-II (BDI-II; Beck et al., 1996, Hautzinger et al., 2006), measuring self-reports of obsessive-compulsive symptoms and depressive symptoms, respectively (see Table 1). A standardized questionnaire to assess blinding effectiveness and potential adverse effects of tDCS was administered at the end of both sessions.

### 2.3 Transcranial direct current stimulation

Stimulation protocol and tDCS montage were based on the procedure used by Reinhart and Woodman (2014) and their current flow model. Direct current was delivered by a battery-driven, constant current stimulator (DC-Stimulator Plus, neuroConn GmbH, Ilmenau, Germany) through two conductive rubber electrodes. The cathodal electrode (5 × 5 cm) was positioned over the pre-SMA (site FCz according to the extended 10–20 system) and the anodal reference electrode (5 × 10 cm) was placed on the right cheek along the mandibular ramus plane. The electrodes were encased in saline-soaked (0.9% NaCl) sponges and fixed to the head with rubber straps.

In the active tDCS condition, a direct current of 1.5 mA was administered for 20 min with a ramp-up and ramp-down period of 30 s. In the sham condition, the current was applied for only 40 s with a ramp-up and ramp-down phase of 30 s at the beginning of the 20 min period. This sham protocol was used for blinding purposes to induce the same transient tingling sensation as experienced with active stimulation. During tDCS, participants were asked to remain seated and relaxed and were allowed to read provided magazines which had been selected for their neutral content (e.g., documentary magazines with a focus on nature and technology). The stimulation was well tolerated by all participants, with the most frequent adverse effects being transient tingling, itching, and burning sensation. Intensity of adverse effects did not differ significantly between active and sham tDCS (all *p* ≥ .545, paired *t* tests with false discovery rate correction). Post-experimental questioning confirmed that participants could not distinguish between active and sham tDCS, χ^2^(2) = 0.46, *p* = .796.

### 2.4 Task

Following the stimulation, participants performed an arrow version of the flanker task (Eriksen & Eriksen, 1974). The software Presentation (Neurobehavioral Systems, Albany, CA, USA) was used for stimulus presentation and response recording. Stimuli consisted of five vertically arranged arrows pointing to the left or right that were presented in white color against a black background. Participants were instructed to indicate the direction of the central target arrow as quickly and accurately as possible by button press. The central target arrow was flanked by arrows pointing in the same direction (50% congruent trials) or by arrows pointing in the opposite direction (50% incongruent trials). Stimulus congruency and arrow direction were varied pseudorandomly within each block of trials.

Each trial started with a fixation cross presented for a random interval between 200 and 1200 ms. Afterwards, the arrows were displayed for 100 ms, followed by presentation of a fixation cross for 700 ms, resulting in a response window of 800 ms after stimulus onset. Participants completed 20 practice trials, presented before tDCS was administered to reduce the time interval between stimulation and start of the task. The task consisted of 480 trials presented in six blocks separated by short breaks. After each block, performance-based feedback was provided. If the error rate in a block was low (≤ 5%), participants were instructed to respond faster; if the error rate was high (≥ 15%), they were instructed to respond more accurately. Otherwise, participants were reminded to keep responding quickly and accurately. Total task duration was approximately 15 min.

### 2.5 EEG recording and preprocessing

The EEG was recorded from 25 Ag/AgCl electrodes mounted in an elastic cap (EASYCAP GmbH, Herrsching, Germany) and positioned according to the extended 10–20 system (Fp1, Fp2, F9, F7, F3, Fz, F4, F8, F10, FC1, FCz, FC2, T7, C3, Cz, C4, T8, CPz, P7, P3, Pz, P4, P8, O1, O2). All electrodes were referenced online to the right mastoid and grounded to an electrode placed below T1. The electrooculogram was recorded from electrodes placed at the outer canthi of both eyes (F9, F10) and above and below the left eye (Fp1, IO1). Electrode impedances were kept below 5 kΩ. EEG recordings were amplified using a BrainAmp amplifier (BrainProducts, Gilching, Germany) with a band-pass filter of 0.01–250 Hz and digitized at a sampling rate of 1000 Hz.

Offline preprocessing was performed with MATLAB (Version 2019b; The MathWorks, Inc., Natick, MA, USA) using the EEGLAB toolbox (Version 2019.1; Delorme & Makeig, 2004) and the ERPLAB toolbox (Version 8.01; Lopez-Calderon & Luck, 2014). The EEG was filtered using a second-order zero phase-shift Butterworth band-pass filter from 0.1 to 30 Hz (half-amplitude cutoff; 12 dB/octave roll-off) and a notch filter at 50 Hz. Data were rereferenced to the average of the mastoids and downsampled to 500 Hz. Ocular artifacts were corrected by independent component analysis applying the extended infomax algorithm (Jung et al., 2000) as implemented in EEGLAB. To help identify components associated with eye movements, we used the SASICA toolbox (Chaumon et al., 2015).

Data were segmented into response-locked epochs of 1500 ms, including a pre-response interval of 500 ms. The interval from −500 to −300 ms prior to the response served as baseline. We used an early interval for baseline correction since error-related activity may start prior to response onset (Klawohn et al., 2020b), and condition-related differences in the pre-response interval were evident when using a response-proximal baseline. EEG epochs containing artifacts, that is, a voltage change exceeding 50 μV between sample points or 200 μV within an epoch, were rejected. On average, 0.57% (*SD* = 0.96) of trials per participant were removed by the artifact rejection procedure (controls: *M* = 0.30%, *SD* = 0.51, range = 0.00–2.08; OCD: *M* = 0.84%, *SD* = 1.20, range = 0.00–5.42).

Error-trial ERN and correct-trial CRN were quantified as mean amplitudes from 0 to 100 ms post-response at electrode FCz on single-trial level. The Pe was measured as the mean amplitude from 200 to 400 ms after errors at electrode Pz. Component quantification was determined a priori based on the literature (Gehring et al., 2012). We examined the internal consistency of these ERPs using a permutation-based split-half approach (splithalf package, Version 0.7.1; Parsons, 2021) with 5000 random splits and Spearman–Brown correction. Results indicated excellent internal consistency for ERN (*r* = .95, 95% CI [.93, .97]), CRN (*r* = 1.00, 95% CI [.99, 1.00]), and Pe (*r* = .94, 95% CI [.91, .96]).

For a non-preregistered post hoc analysis (see below for more details), we additionally quantified the stimulus-locked P300, a component associated with attention allocation and updating of working memory (for a review, see Polich, 2012). EEG data were segmented into stimulus-locked epochs of 1500 ms starting 500 ms prior to stimulus onset and baseline corrected using the 200-ms pre-stimulus interval. The P300 was measured as the mean amplitude between 300 and 500 ms at electrode CPz.

### 2.6 Statistical analysis

Data were analyzed in R (Version 3.6.1) using linear mixed models (LMMs) on single-trial behavioral and ERP data. Trials were excluded from all analyses if the response time was below 100 ms or above 800 ms (average percentage of excluded trials per participant: *M* = 0.04%, *SD* = 0.10, range = 0.00–0.62), or if no response was made (*M* = 0.64%, *SD* = 0.93, range = 0.00–5.00).

We used LMMs for statistical inference, as they are robust to unbalanced data (Pinheiro & Bates, 2000). This makes mixed-effects modeling an advantageous approach in research on error monitoring since the number of observations entering the analysis is determined by the participant’s performance. Moreover, due to consideration of random slopes, LMMs account for random variance in effect sizes across participants, thereby decreasing the rate of Type I errors for associated fixed effects (Barr et al., 2013, Matuschek et al., 2017).

We analyzed behavioral and ERP measures and tested whether group differences and tDCS effects were present. Group (healthy controls, OCD) and tDCS condition (cathodal, sham) were included as fixed effects in all models. All categorical fixed effects were effect-coded (contrast coefficients −0.5 and 0.5). We determined the random-effects structure for each model based on the procedure proposed by Bates et al. (2015a), starting with the maximal random-effects structure justified by the design, with by-participant random intercepts and random slopes for all fixed factors and (where applicable) their interactions. If required for model convergence, correlation parameters of the random terms were set to zero. Random effects preventing model convergence or explaining zero variance as determined by principal component analysis were removed to avoid overparameterization.

Models were fitted using the lme4 package (Version 1.1-25; Bates et al., 2015b) and *p* values for LMMs were calculated using the Satterthwaite approximation for degrees of freedom as implemented in the lmerTest package (Version 3.1-3; Kuznetsova et al., 2017). The significance level was *p* < .05. We evaluated whether model assumptions were met using the performance package (Version 0.7.1.1; Lüdecke et al., 2021). For final models, we report unstandardized effect sizes (regression coefficients *b*) with 95% confidence intervals, test statistics (*t*/*z* values), and *p* values. Reported estimates were calculated using restricted maximum likelihood estimation. Data and analysis scripts are available at https://osf.io/7z8hj/.

#### 2.6.1 Behavioral data

We analyzed response time data using a LMM with response type (correct, incorrect), group, and tDCS condition as predictors. Response time was log-transformed prior to analysis to meet the assumption of normally distributed residuals. The appropriate transformation was determined using the Box–Cox procedure (Box & Cox, 1964).

For the analysis of PES, we fitted a LMM on single-trial values that were calculated as the response time difference between correct responses that directly preceded and followed an error. This PES quantification results in a measure that is not confounded by fluctuations in motivation or response caution over time (Dutilh et al., 2012). In this analysis, we considered only error trials that were preceded by at least two correct trials and followed by at least one correct trial. Model estimates of the LMM on PES directly reflect mean differences in milliseconds.

Response accuracy was analyzed using a binomial generalized linear mixed model (GLMM). For the GLMM on accuracy, estimates reflect odds ratios for a correct response and *p* values were obtained using Wald *Z* tests.

#### 2.6.2 ERP data

We fitted a LMM with response-related negativity (corresponds to ERN for incorrect trials and CRN for correct trials) as dependent variable to examine the presence of an overall tDCS effect on electrophysiological correlates of performance monitoring. Both correct and incorrect trials were included in this analysis. We entered group, tDCS condition, and response type as predictors. Additionally, analyses were conducted separately for incorrect and correct trials, such that separate LMMs were specified with ERN, Pe, and CRN as dependent variables. In accordance with the preregistration, these separate models for ERN and CRN were specified in addition to the overall model for the response-related negativity to allow comparison with previously reported results on the ERN alone.

We performed additional control analyses to examine whether tDCS effects on ERPs were affected by psychotropic medication. In these analyses, we accounted for medication status by respecifying the fixed effect *group* as a factor with three levels (control participants, medicated patients with OCD, unmedicated patients with OCD), which was coded using sliding difference contrasts.

In an additional (non-preregistered) analysis, we included the within-participant *z*-standardized single-trial P300 amplitude as a covariate into the analysis of the response-locked ERPs. Thereby, we aimed to control for variation in the P300 amplitude since response-locked ERPs often overlap with this stimulus-locked positivity, which makes inferences about effects on response-locked components more difficult (Hajcak et al., 2004, Klawohn et al., 2020c, Meyer et al., 2017). Visual inspection of the ERPs suggested that this also holds true for our data. Specifically, we aimed to control for tDCS-related P300 differences because an exploratory analysis indicated that the P300 was modulated by tDCS (see Table S5 in the supplemental material). Since including a covariate in the models results in a multiple testing scenario regarding the primary hypothesis addressing the effect of cathodal tDCS on ERN amplitude, the Holm–Bonferroni correction was applied for the main effect of tDCS on the ERN.

## 3 Results

### 3.1 Demographic and clinical characteristics

Table 1 summarizes demographic and clinical characteristics of the two groups along with results of group comparisons. The groups did not differ in gender, age, and level of education. As expected, patients with OCD reported significantly higher severity of obsessive-compulsive and depressive symptoms compared to control participants.

### 3.2 Behavioral results

Descriptive statistics for behavioral performance in the cathodal and sham tDCS condition for both groups are reported in Table 2. Full model results of the (G)LMMs on behavioral data are provided in the supplemental material (see Tables S1 and S2).

**Table 2:** Behavioral and Event-Related Potential (ERP) Measures in the Groups of Patients With Obsessive-Compulsive Disorder (OCD) and Healthy Control (HC) Participants After Sham and Cathodal Transcranial Direct Current Stimulation (tDCS).

Measure	Sham tDCS			Cathodal tDCS	
	Patients with OCD	HC participants		Patients with OCD	HC participants
	*M* [95% CI]	*M* [95% CI]		*M* [95% CI]	*M* [95% CI]
*Behavioral measures*					
RT correct (ms)	416 [415, 418]	401 [400, 403]		411 [410, 412]	403 [401, 404]
RT error (ms)	346 [341, 352]	341 [335, 346]		345 [340, 350]	335 [330, 340]
PES (ms)	40 [28, 51]	29 [20, 39]		39 [29, 50]	32 [21, 42]
Accuracy (%)	94.68 [94.14, 95.21]	93.24 [92.64, 93.84]		94.14 [93.57, 94.70]	93.51 [92.92, 94.10]

*ERP measures*					
ERN (μV)	−3.81 [−4.69, −2.93]	−1.33 [−2.17, −0.49]		−3.57 [−4.44, −2.70]	−0.22 [−1.09, 0.65]
CRN (μV)	4.37 [4.18, 4.56]	7.03 [6.84, 7.23]		5.49 [5.30, 5.69]	7.17 [6.96, 7.37]
Pe (μV)	8.64 [7.76, 9.51]	8.02 [7.29, 8.75]		9.60 [8.81, 10.40]	8.92 [8.16, 9.67]

*Note.* Confidence intervals (CIs) are adjusted for within-participant designs (Morey, 2008). Means and CIs were calculated from single-trial data. Error-related negativity (ERN) and correct-response negativity (CRN) were quantified as mean amplitude from 0 to 100 ms at electrode FCz. Error positivity (Pe) was quantified as mean amplitude from 200 to 400 ms at electrode Pz. RT = response time; PES = post-error slowing.

#### 3.2.1 Response time

Response time analysis revealed a significant main effect of response type (*b* = −0.17, 95% CI [−0.19, −0.15], *t* = −16.46, *p* < .001), indicating that incorrect responses were faster than correct responses. Response time did not differ significantly between groups (*b* = 0.02, 95% CI [−0.02, 0.06], *t* = 0.99, *p* = .328) or tDCS conditions (*b* = −0.00, 95% CI [−0.02, 0.01], *t* = −0.53, *p* = .599) and there was no significant interaction between any of the factors (all |*t*| ≤ 1.68, *p* ≥ .099).

#### 3.2.2 Post-error slowing

The LMM on PES yielded a significant intercept, reflecting that participants slowed down after error commission (*b* = 35.71, 95% CI [29.40, 42.02], *t* = 11.09, *p* < .001). The groups did not differ significantly in PES (*b* = 9.59, 95% CI [−3.03, 22.21], *t* = 1.49, *p* = .142). There was no significant main effect of tDCS condition (*b* = 1.08, 95% CI [−8.73, 10.89], *t* = 0.22, *p* = .830) and no interaction between group and tDCS condition (*b* = −3.08, 95% CI [−22.70, 16.54], *t* = −0.31, *p* = .760).

#### 3.2.3 Response accuracy

Results of the GLMM indicated that there was no significant difference in response accuracy between groups (odds ratio = 1.18, 95% CI [0.96, 1.44], *z* = 1.54, *p* = .123) or tDCS conditions (odds ratio = 0.97, 95% CI [0.88, 1.07], *z* = −0.56, *p* = .573) and no significant interaction between group and tDCS condition (odds ratio = 0.84, 95% CI [0.70, 1.02], *z* = −1.71, *p* = .087).

### 3.3 ERP results

**Fig. 1:**
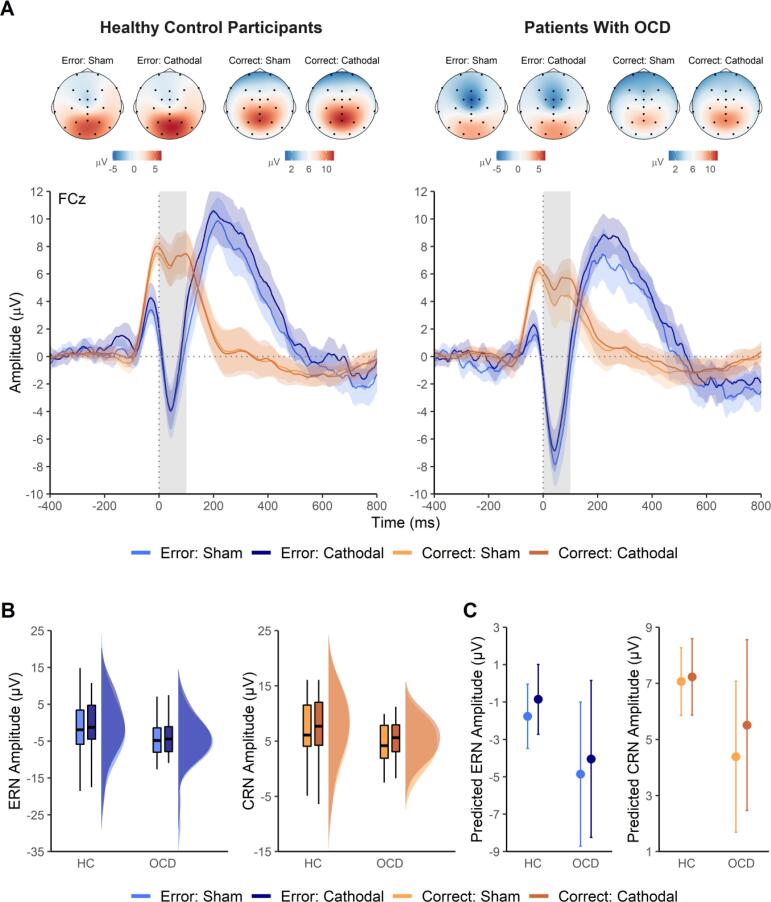
Effects of transcranial direct current stimulation (tDCS) on the error-related negativity (ERN) and the correct-response negativity (CRN) in patients with obsessive-compulsive disorder (OCD) and healthy control (HC) participants. **(A)** Response-locked grand average waveforms with 95% confidence intervals (CIs) for correct and incorrect responses at electrode site FCz in the sham and cathodal tDCS condition for patients with OCD and HC participants, along with topographies of ERN and CRN (0–100 ms). Gray-shaded areas in the waveform plots indicate the time window used for ERN and CRN quantification. **(B)** ERN and CRN mean amplitude values per group and tDCS condition presented as boxplots and probability density plots based on raw data. **(C)** Predicted ERN and CRN mean amplitude values per group and tDCS condition calculated as partial effects from linear mixed models. Error bars represent 95% CIs. **(A–C)** The plots were generated using the packages eegUtils (Version 0.5.0; Craddock, 2020), raincloudplots (Version 0.2.0; Allen et al., 2021), and sjPlot (Version 2.8.6; Lüdecke, 2020). Note differences in y-axis scales between graphs in panels B and C.

Response-locked ERPs for both groups in the sham and cathodal tDCS condition are displayed in Fig. 1. Mean ERP amplitude values are presented in Table 2. In Table 3, we provide model estimates from the LMM analysis of the ERPs. In these analyses, model estimates directly reflect mean differences in microvolts. Note that for negative components, such as the ERN and CRN, negative estimates indicate an increase in amplitude, whereas positive estimates indicate a decrease.

**Table 3:** Results of the Linear Mixed Models (LMMs) Predicting the Error-Related Negativity (ERN), Correct-Response Negativity (CRN), and Error Positivity (Pe) Amplitude as a Function of Stimulation Condition (Cathodal − Sham) and Group (OCD – Healthy Controls).

	ERN				CRN				Pe			
*Fixed effects*	*b*	95% CI	*t*	*p*	*b*	95% CI	*t*	*p*	*b*	95% CI	*t*	*p*
Intercept	−2.88	[−4.60, −1.16]	−3.29	**.002**	6.05	[4.84, 7.25]	9.84	**< .001**	9.28	[8.03, 10.53]	14.56	**< .001**
Stimulation	0.86	[0.02, 1.70]	2.00	.052 ^a^	0.65	[0.06, 1.24]	2.18	**.034**	0.94	[0.22, 1.65]	2.57	**.013**
Group	−3.14	[−6.58, 0.30]	−1.79	.079	−2.20	[−4.61, 0.21]	−1.79	.079	0.42	[−2.08, 2.91]	0.33	.745
Stimulation × Group	−0.10	[−1.78, 1.59]	−0.11	.911	0.96	[−0.21, 2.13]	1.60	.114	0.55	[−0.87, 1.98]	0.76	.451
												
*Random effects*	*SD*				*SD*				*SD*			
Participants (intercept)	6.41				4.59				4.63			
Stimulation	1.72				2.14				1.59			
Residual	9.93				9.58				8.07			

*Note.* The maximal random-effects structure was used in all models. Results are based on 3244 and 49849 observations for ERN/Pe and CRN, respectively. Statistically significant *p* values (*p* < .05) are shown in bold. OCD = obsessive-compulsive disorder; CI = confidence interval.

^a^ Holm–Bonferroni-adjusted *p* value is reported. Since this *p* value is the largest in the set of comparisons, the corrected *p* value is equal to the uncorrected *p* = .052.

#### 3.3.1 Response-related negativity

The LMM on the response-related negativity across correct and incorrect responses revealed a main effect of response type (*b* = −8.96, 95% CI [−10.32, −7.60], *t* = −12.90, *p* < .001), with more negative amplitudes for errors (ERN) than for correct responses (CRN). A trend for an enhanced response-related negativity in patients with OCD compared to control participants was observed (*b* = −2.64, 95% CI [−5.27, −0.01], *t* = −1.97, *p* = .054). Crucially, we found that the response-related negativity was reduced (i.e., less negative) after cathodal tDCS relative to sham tDCS, as evidenced by a main effect of tDCS condition (*b* = 0.70, 95% CI [0.12, 1.28], *t* = 2.37, *p* = .022). There was no significant interaction between any of the factors (all |*t*| ≤ 0.95, *p* ≥ .349). Full model results of the LMM are presented in the supplemental material (see Table S3).

#### 3.3.2 Error-related negativity

In the analysis of the ERN, the main effect of group did not reach statistical significance, but a trend for an enhanced ERN amplitude in patients with OCD relative to healthy control participants was observed (see Table 3). The same trend was evident when solely the baseline ERN (i.e., the sham tDCS condition) was considered (*b* = −3.22, 95% CI [−6.82, 0.38], *t* = −1.76, *p* = .085). Moreover, there was a statistical trend (*p* = .052, Holm–Bonferroni-adjusted) toward a reduced ERN amplitude after cathodal tDCS relative to sham tDCS (see Table 3). No significant interaction between group and tDCS condition was found, indicating that there was no evidence that the effect of tDCS on ERN amplitude was larger in patients with OCD than in healthy participants.

#### 3.3.3 Correct-response negativity

The LMM on the CRN yielded a trend for a main effect of group, such that patients with OCD showed an enhanced CRN amplitude compared to control participants (see Table 3). When evaluating group differences solely in the baseline CRN (i.e., in the sham tDCS condition), this effect reached significance (*b* = −2.68, 95% CI [−5.14, −0.23], *t* = −2.14, *p* = .037). In addition, a significant main effect of tDCS condition revealed that the CRN amplitude was significantly smaller after cathodal tDCS relative to sham tDCS (see Table 3). There was no significant interaction between group and tDCS condition.

#### 3.3.4 Error positivity

Analysis of the Pe amplitude indicated that this component was increased after cathodal tDCS relative to sham tDCS, as evidenced by a significant main effect of tDCS condition (see Table 3 and Fig. S1 in the supplemental material). No significant main effect of group and no interaction between group and tDCS condition were observed.

#### 3.3.5 Controlling for effects of psychotropic medication

We accounted for possible confounding effects of psychotropic medication by respecifying the fixed effect *group* as a factor with three levels (healthy controls, medicated patients with OCD, unmedicated patients with OCD). Results remained unchanged, with a trend for a main effect of tDCS on ERN amplitude (*b* = 0.86, 95% CI [−0.05, 1.77], *t* = 1.85, *p* = .070) and a significant main effect of tDCS on CRN (*b* = 0.83, 95% CI [0.21, 1.45], *t* = 2.62, *p* = .011) and Pe amplitude (*b* = 0.96, 95% CI [0.23, 1.68], *t* = 2.59, *p* = .012). Detailed results of the control analyses are available in the supplemental material (see Table S4).

#### 3.3.6 Controlling for P300 amplitude

When including the P300 as a covariate in the LMMs on ERN, CRN, and Pe amplitude, a significant main effect of the P300 was observed in all models (all |*t*| ≥ 31.74, *p* < .001). The effect of tDCS on CRN and Pe remained significant (CRN: *b* = 0.67, 95% CI [0.09, 1.26], *t* = 2.25, *p* = .028; Pe: *b* = 0.83, 95% CI [0.07, 1.60], *t* = 2.13, *p* = .038). Importantly, the tDCS-induced reduction in ERN amplitude, previously present as a statistical trend, was now significant (*b* = 0.91, 95% CI [0.18, 1.64], *t* = 2.45, *p* = .034, Holm–Bonferroni-adjusted). The same applies to the group difference in ERN amplitude, which now also reached significance (*b* = −2.99, 95% CI [−5.83, −0.14], *t* = −2.06, *p* = .044). Detailed results of the analysis exploring tDCS effects on the P300 and the analyses including P300 as a covariate are available in the supplemental material (see Tables S5 and S6).

#### 3.3.7 Test for statistical equivalence

Since the tDCS-induced ERN reduction emerged only as a statistical trend in the main analysis, we further examined this effect using the two one-sided tests procedure for equivalence testing (Lakens et al., 2018) in a non-preregistered post hoc analysis. This procedure allowed us to test whether the ERN amplitude was statistically equivalent in the sham and cathodal tDCS condition, or whether our study was just not sufficiently sensitive to clearly detect the stimulation effect. While traditional null hypothesis significance testing can provide support only for the presence of an effect, equivalence testing allows to test whether a meaningful effect is absent, that is, whether the presence of an effect at least as extreme as a smallest effect size of interest (SESOI) can be rejected. As recommended by Simonsohn (2015) for studies building on previous work, we defined the SESOI as the effect size that the study by Reinhart and Woodman (2014) had 33% power to detect. This approach tests for the presence of an effect that a previous study could have meaningfully examined. On the basis of this approach, we set the SESOI for equivalence bounds to Cohen’s *d_z_* = 0.38, which corresponds to an ERN amplitude difference of 1.34 μV between cathodal and sham tDCS. The equivalence test was not significant, *t*(44.18) = −1.12, *p* = .134, indicating that the ERN amplitude in the cathodal condition was not statistically equivalent to that in the sham condition. Thus, we cannot reject the presence of an effect as large or larger than 1.34 μV.

Taken together, based on results from null hypothesis testing and equivalence testing, we can neither reliably conclude that the effect of cathodal tDCS on ERN amplitude is different from zero (no statistical significance, only a statistical trend), nor that an effect that can be considered meaningful is absent (no statistical equivalence). Notably, when controlling for the P300 amplitude, a significant effect of tDCS on the ERN was evident.

## 4 Discussion

In this study, we investigated whether non-invasive brain stimulation targeting the pre-SMA modulates error monitoring in patients with OCD and healthy individuals. As predicted, cathodal tDCS reduced the ERN amplitude compared to sham tDCS, albeit this effect was only marginally significant. Furthermore, cathodal tDCS reduced the CRN amplitude and increased the Pe amplitude. Contrary to our predictions, these ERP modulations were not accompanied by behavioral changes, such as an increased error rate or reduced PES. Moreover, we found no evidence that the stimulation effect was more pronounced in the patient group compared to the control group. Regarding baseline ERP group differences, we observed enhanced ERN (at trend level) and CRN amplitudes in the patient group relative to the control group.

Even though our data did not yield strong evidence of a tDCS effect on ERN amplitude, the findings support the notion that cathodal tDCS has promising potential to attenuate error monitoring across healthy individuals and patients with OCD. Beyond the statistical trend in the expected direction observed in the main analysis, results of the equivalence test indicated that the ERN amplitude in the cathodal condition was not statistically equivalent to that in the sham condition. Hence, we cannot reject the presence of an effect that can still be considered meaningful. In addition, when controlling for variation in the stimulus-locked P300, that often overlaps with the ERN, a significant tDCS-induced ERN reduction became evident. In their entirety, these results indicate that a single session of cathodal tDCS reduces the ERN amplitude in healthy individuals and patients with OCD, but the effect appears to be small (effect size in this study: 0.86 μV, 95% CI [0.02, 1.70]) and more data are needed to draw definite conclusions.

Effects of tDCS on the ERN are possibly subject to variations in experimental design and tDCS protocol, consistent with the fact that previous studies in healthy individuals yielded heterogeneous results. Reinhart and Woodman (2014) reported that cathodal tDCS at 1.5 mA over the pre-SMA (electrode site FCz) reduced the ERN in a stop-signal task. In contrast, Bellaïche et al. (2013), delivering cathodal tDCS at 1 mA over the medial prefrontal cortex (electrode site Fpz), found no effects on the ERN in a flanker task. Relatively small sample sizes may have contributed to the inconsistency in findings.

Although the present study was better powered than the study by Reinhart and Woodman (2014) and used an almost identical tDCS protocol, we did not observe such a robust ERN reduction. This suggests that the effect could be weaker than originally reported. Alternatively, the discrepancy between findings may be due to differences in experimental tasks (target discrimination task with stop signals and learning demands vs. flanker task), given that there are task-specific effects on the ERN (Riesel et al., 2013). Additionally, inconsistent findings may result from heterogeneity in individual characteristics. In particular, factors such as age, hormonal and neurotransmitter levels, baseline cortical activity, and skull and cortical morphology seem to influence the response to electrical cortical stimulation (Krause & Cohen Kadosh, 2014). Moreover, findings may be affected by the time interval between stimulation and ERN assessment. Verveer et al. (2021) observed that effects of high-definition tDCS on ERN amplitude occurred 30 min after the stimulation. This accords with evidence showing that modulation of cortical excitability by tDCS reaches its maximum about 30 min after the stimulation (Kuo et al., 2013). In summary, optimal experimental designs and tDCS protocols still need to be determined.

Nevertheless, our finding of ERN reduction after a single session of tDCS lays promising groundwork for future studies to examine whether repeated tDCS application normalizes overactive error monitoring in OCD. Since this technique is time- and cost-efficient, using tDCS to target aberrant error monitoring could be a viable adjunct or even alternative treatment strategy for individuals with OCD or anxiety disorders, or a prevention strategy for populations at risk for such disorders. To date, only few studies directly targeted overactive error monitoring in OCD. Experimental manipulations such as dual-task demands (Klawohn et al., 2016) or training procedures such as attentional bias modification (Klawohn et al., 2020a, Tan et al., 2021) have been found to temporarily reduce the ERN in patients with OCD. It is an open question, however, whether such approaches have the potential to induce long-lasting effects. Considering that after-effects of prolonged tDCS protocols presumably involve synaptic plasticity (Bikson et al., 2019), this technique may be particularly promising for inducing long-lasting effects. Importantly, there is evidence that stimulation effects accumulate over repeated administration of tDCS, thereby increasing modulatory efficacy (Alonzo et al., 2012, Ho et al., 2016). Thus, further research is needed to elucidate whether repeated application of tDCS induces a more pronounced and sustained ERN attenuation and whether such ERN reduction impacts clinical outcomes.

The present findings give a hint of the possible underlying mechanism by which inhibitory pre-SMA stimulation may reduce OCD symptoms as observed in previous studies (for reviews, see e.g., Brunelin et al., 2018, Rapinesi et al., 2019). Our findings support the notion that a reduction in overactive error monitoring might be involved. Specifically, cathodal tDCS may reduce pre-SMA hyperactivity, which is considered to play a relevant role in OCD pathophysiology (de Wit et al., 2012) and to underlie overactive error monitoring in OCD (Grützmann et al., 2016). Normalization of pre-SMA activity could thus reduce overactive error monitoring and modify associated pathophysiological processes, thereby reducing symptom severity.

Contrary to our predictions, we found no evidence that the tDCS effect on ERN amplitude was larger in patients with OCD than in healthy participants. This is in contrast to prior research indicating that experimental manipulations induced greater ERN reduction in patients with OCD than in healthy individuals (Klawohn et al., 2016, Klawohn et al., 2020a). Similarly, an intervention targeting error sensitivity decreased the ERN more effectively in individuals with larger baseline ERN (Meyer et al., 2020). Unlike these approaches, tDCS may be effective in modulating error monitoring over the entire range of ERN magnitude. This notion is supported by the finding that anodal tDCS was equally effective in enhancing the ERN in healthy individuals and patients with schizophrenia, who typically have smaller ERN amplitudes (Reinhart et al., 2015). However, it should be noted that ERN group differences emerged only as a statistical trend in our sample. Thus, it cannot be excluded that modulatory effects are indeed larger at the upper end of ERN magnitude range, but that this could not be detected in the present study.

Our findings further indicated that cathodal tDCS increased the Pe amplitude, consistent with previously reported effects of inhibitory pre-SMA stimulation by cathodal tDCS (Reinhart & Woodman, 2014) and low-frequency rTMS (Rollnik et al., 2004). Both studies interpreted the Pe enhancement as an indicator of an increased affective response to errors, in line with the interpretation of the Pe as an index of the emotional significance of errors (Falkenstein et al., 2000, Overbeek et al., 2005) and the involvement of the medial frontal cortex in affective processing (Shackman et al., 2011). Alternatively, and in our view more plausibly, results can be interpreted within the dual mechanisms of control framework (Braver, 2012) that postulates two modes of cognitive control: proactive control (i.e., preparatory control by allocation of attention resources to enable optimal response to upcoming demanding events) and reactive control (i.e., transient engagement of control upon detection of conflict or errors). In the context of this theory, the Pe enhancement may reflect a compensatory increase in proactive control via adjustments in attentional engagement. This compensatory effort may be employed to maintain adequate task performance despite the tDCS-induced ERN reduction that possibly indicates a reduced engagement of reactive control. Indeed, the ERN has been associated with reactive control and the Pe with proactive control in previous studies (e.g., Boksem et al., 2006, Larson et al., 2016, Moser et al., 2013).

In addition to the modulation of neural responses to errors, we found that tDCS also affected the CRN amplitude. A different pattern of results was reported by Reinhart and Woodman (2014), who observed that cathodal tDCS reduced the ERN but had no effect on the CRN. In contrast, our results suggest that the stimulation affected response monitoring processes across action outcomes rather than error-specific processes. In general, literature on the CRN is relatively scarce and inconsistent. The functional significance of this component and how it is affected by factors such as task difficulty and individual differences is not well understood (e.g., Endrass and Ullsperger, 2014, Riesel et al., 2015b). Notably, prior research indicates that both error-specific and general monitoring processes are overactive in OCD (Klawohn et al., 2014), consistent with several studies reporting that both ERN and CRN are increased in OCD (for a review, see Michael et al., 2021). Accordingly, the lack of selectivity of tDCS effects on response monitoring processes might be no disadvantage in the use of this technique for normalizing overactive performance monitoring in OCD.

In our study, ERP modulations were not accompanied by behavioral changes. In fact, prior findings of tDCS effects on behavioral indices of performance monitoring have been mixed. Reinhart and Woodman (2014) reported that cathodal tDCS increased error rates and reduced PES. In contrast, and consistent with our findings, two other studies found that tDCS effects on neural responses to errors did not translate into behavioral changes (Bellaïche et al., 2013, Verveer et al., 2021). One possible explanation for the absence of behavioral effects despite the presence of ERP effects is that behavioral adjustments are possibly not directly linked to medial frontal brain activity and electrophysiological correlates of performance monitoring (Danielmeier and Ullsperger, 2011, Ullsperger et al., 2014). Neural mechanisms underlying post-error behavioral adjustments such as PES are still not fully understood (Ullsperger et al., 2014). Indeed, several studies have failed to find an association between ERN and PES (for a meta-analysis, see Cavanagh & Shackman, 2015), and ERN enhancement in OCD is observed in most studies in the absence of behavioral differences (Endrass & Ullsperger, 2014). Moreover, it is possible that the lack of effects at the behavioral level can be attributed to the performance-based feedback that was provided to obtain a sufficient number of errors. Alternatively, ERP modulations after a single tDCS session may just not have been substantial enough to impact behavior. Importantly, these results shed light on the direction of the causal chain that remains unresolved when both neural and behavioral effects are present (Reinhart & Woodman, 2014). Our findings indicate that tDCS affects neural correlates of performances monitoring directly rather than indirectly by inducing behavioral changes, such as increasing error rates.

Some limitations should be considered when interpreting the results. First, the findings are limited to the effects of one session of tDCS. In this proof-of-concept study, we sought to provide an initial assessment of modulatory effects of tDCS on error monitoring in OCD. Repeated application of tDCS may be required to induce marked and sustained effects on error monitoring and possibly reduce symptom severity. Even though previous findings suggest that modulation of neural correlates of error monitoring could be of relevance to the treatment of OCD or may impact psychological mechanisms that increase risk for psychopathology (Banica et al., 2021, Riesel et al., 2021, Sildatke et al., 2022), more research is needed to evaluate the therapeutic relevance of ERN modulation. Thus, an avenue for future research is to examine whether long-lasting changes in the ERN can be induced (e.g., by repeated tDCS application), and to what extent modulating the ERN may relate to subsequent change at the symptom level.

Second, the mechanism by which tDCS over the pre-SMA modulates performance monitoring remains to be further elucidated. Combination with functional neuroimaging may reveal direct effects on brain activation patterns. Such insights may contribute to a more targeted use of tDCS in therapeutic contexts.

Finally, the applied tDCS protocol was based on a previous study that found this protocol to be effective in modulating error monitoring in healthy individuals (Reinhart & Woodman, 2014). It is possible that in clinical populations, different protocols would be even more effective in normalizing error monitoring. Future studies could combine non-invasive stimulation with neuronavigation methods to precisely locate cortical targets based on individual anatomy or employ neuroimaging during symptom provocation to reveal neural targets directly associated with obsessive-compulsive symptomatology. Moreover, further investigation is needed to define the characteristics of patients who would benefit the most from such intervention approaches. For instance, prior studies examining tDCS effects on OCD symptom severity included only treatment-resistant patients (for reviews, see e.g., Brunelin et al., 2018, Rapinesi et al., 2019), limiting the generalizability of the findings. In sum, optimal stimulation parameters and relevant factors influencing the response to tDCS still need to be determined.

In conclusion, the present findings indicate that cathodal tDCS targeting the pre-SMA modulates neural correlates of performance monitoring and may have the potential to attenuate overactive performance monitoring in OCD. Our results provide evidence that a single session of cathodal tDCS reduces ERN and CRN amplitudes across healthy individuals and patients with OCD. Even though more data are needed to draw definite conclusions, these findings provide a useful basis for future research that may determine whether repeated application of tDCS rebalances abnormal activation patterns and normalizes overactive error signals in OCD in the long term, thereby potentially alleviating obsessive-compulsive symptoms. Accordingly, the present results substantiate the assumed role of the ERN as a potential target for novel intervention and prevention strategies. In particular, tDCS might be a promising strategy due to its non-invasive character, its time- and cost-efficiency, and its potential to induce long-lasting effects.

## CRediT authorship contribution statement

**Luisa Balzus:** Conceptualization, Methodology, Formal analysis, Investigation, Data curation, Writing - original draft, Visualization. **Julia Klawohn:** Conceptualization, Methodology, Writing - review & editing, Supervision. **Björn Elsner:** Writing - review & editing. **Sein Schmidt:** Methodology, Writing - review & editing. **Stephan A. Brandt:** Methodology, Resources, Writing - review & editing. **Nobert Kathmann:** Conceptualization, Methodology, Resources, Writing - review & editing, Supervision, Project administration, Funding acquisition.

## Declaration of Competing Interest

The authors declare that they have no known competing financial interests or personal relationships that could have appeared to influence the work reported in this paper.

## Data Availability

This study was registered in the German Clinical Trials Register (ID: DRKS00016807). Preregistration, data, and code for all analyses are available at https://osf.io/7z8hj/.
