# Supplementary material for: Non-invasive brain stimulation modulates neural correlates of performance monitoring in patients with obsessive-compulsive disorder
Source: Neuroimage Clin. 2022 Jul 14;35:103113. doi: 10.1016/j.nicl.2022.103113 (PMC9421486; doi:10.1016/j.nicl.2022.103113)
Supplement: Supplementary data 1 [file mmc1.docx]

**Non-Invasive Brain Stimulation Modulates Neural Correlates of Performance Monitoring in Patients With Obsessive-Compulsive Disorder**

Luisa Balzus^a, b^, Julia Klawohn^a^, Björn Elsner^a^, Sein Schmidt^c^, Stephan A. Brandt^c^, and Norbert Kathmann^a^

^a^ Humboldt-Universität zu Berlin, Department of Psychology, Berlin, Germany

^b^ Humboldt-Universität zu Berlin, Berlin School of Mind and Brain, Berlin, Germany

^c^ Charité – Universitätsmedizin Berlin, Department of Neurology, Berlin, Germany

Correspondence: [luisa.balzus@alumni.hu-berlin.de](mailto:luisa.balzus@alumni.hu-berlin.de)

**Supplemental Material**

In this supplemental material, we first provide full model results of the linear mixed models (LMMs) on behavioral data and the response-related negativity (Table S1 to S3), and depict effects of transcranial direct current stimulation (tDCS) on the error positivity (Pe; Fig. S1). We then report results from control analyses examining the effect of psychotropic medication on event-related potentials (Table S4). Finally, we report results from non-preregistered post hoc analyses, exploring effects of tDCS on P300 amplitude (Table S5) and including the P300 as a covariate in the LMMs on the error-related negativity (ERN), the correct-response negativity (CRN), and the Pe (Table S6).

**Table S1**

*Results of the Linear Mixed Model (LMM) Predicting Response Time as a Function of Stimulation Condition (Cathodal* − *Sham), Group (OCD* − *Healthy Controls), and Response Type (Incorrect* − *Correct)*

| **Fixed effects** | *b* | 95% CI | *t* | *p* |
| --- | --- | --- | --- | --- |
| Intercept | 5.91 | [5.89, 5.93] | 568.05 | **< .001** |
| Stimulation | −0.00 | [−0.02, 0.01] | −0.53 | .599 |
| Group | 0.02 | [−0.02, 0.06] | 0.99 | .328 |
| Response type | −0.17 | [−0.19, −0.15] | −16.46 | **< .001** |
| Stimulation × Group | 0.00 | [−0.03, 0.03] | 0.00 | 1.000 |
| Stimulation × Response type | 0.00 | [−0.02, 0.02] | 0.14 | .890 |
| Group × Response type | −0.02 | [−0.06, 0.02] | −0.80 | .426 |
| Stimulation × Group × Response type | 0.03 | [−0.01, 0.07] | 1.68 | .099 |
| **Random effects** | *SD* |  |  |  |
| Participants (intercept) | 0.08 |  |  |  |
| Stimulation | 0.05 |  |  |  |
| Response type | 0.07 |  |  |  |
| Stimulation × Response type | 0.06 |  |  |  |
| Residual | 0.17 |  |  |  |

*Note.* The maximal random-effects structure was used in the model. Results are based on 53,093 observations. Estimates are on the log scale. Statistically significant *p* values (*p* < .05) are shown in bold. OCD = obsessive-compulsive disorder; CI = confidence interval.

**Table S2**

*Results of the Linear Mixed Model (LMM) Predicting Post-Error Slowing (PES) and of the Binomial Generalized Linear Mixed Model (GLMM) Predicting Response Accuracy as a Function of Stimulation Condition (Cathodal* − *Sham) and Group (OCD* − *Healthy Controls)*

|  | PES | | | |  | | Accuracy | | | |
| --- | --- | --- | --- | --- | --- | --- | --- | --- | --- | --- |
| **Fixed effects** | *b* | 95% CI | *t* | *p* | | OR | | 95% CI | *z* | *p* |
| Intercept | 35.71 | [29.40, 42.02] | 11.09 | **< .001** | | 16.45 | | [14.84, 18.24] | 53.10 | **< .001** |
| Stimulation | 1.08 | [−8.73, 10.89] | 0.22 | .830 | | 0.97 | | [0.88, 1.07] | −0.56 | .573 |
| Group | 9.59 | [−3.03, 22.21] | 1.49 | .142 | | 1.18 | | [0.96, 1.44] | 1.54 | .123 |
| Stimulation × Group | −3.08 | [−22.70, 16.54] | −0.31 | .760 | | 0.84 | | [0.70, 1.02] | −1.71 | .087 |
| **Random effects** | *SD* |  |  |  | | *SD* | |  |  |  |
| Participants (intercept) | 19.17 |  |  |  | | 0.37 | |  |  |  |
| Stimulation | 23.80 |  |  |  | | 0.24 | |  |  |  |
| Residual ^a^ | 95.82 |  |  |  | |  | |  |  |  |

*Note.* The maximal random-effects structure was used in both models. Results are based on 2,622 and 53,093 observations for PES and accuracy, respectively. Estimates of the GLMM on accuracy indicate the odds ratios for a correct response. Statistically significant *p* values (*p* < .05) are shown in bold. OCD = obsessive-compulsive disorder; CI = confidence interval; OR = odds ratio.

^a^ Binomial GLMMs do not provide a direct estimate of residual variance.

**Table S3**

*Results of the Linear Mixed Model (LMM) Predicting the Response-Related Negativity Amplitude as a Function of Stimulation Condition (Cathodal* − *Sham), Group (OCD* − *Healthy Controls), and Response Type (Incorrect* − *Correct)*

| **Fixed effects** | *b* | 95% CI | *t* | *p* |
| --- | --- | --- | --- | --- |
| Intercept | 1.57 | [0.25, 2.88] | 2.33 | **.023** |
| Stimulation | 0.70 | [0.12, 1.28] | 2.37 | **.022** |
| Group | −2.64 | [−5.27, −0.01] | −1.97 | .054 |
| Response type | −8.96 | [−10.32, −7.60] | −12.90 | **< .001** |
| Stimulation × Group | 0.52 | [−0.64, 1.68] | 0.88 | .383 |
| Stimulation × Response type | 0.10 | [−0.81, 1.01] | 0.22 | .830 |
| Group × Response type | −0.88 | [−3.60, 1.84] | −0.63 | .528 |
| Stimulation × Group × Response type | −0.88 | [−2.69, 0.94] | −0.95 | .349 |
| **Random effects** | *SD* |  |  |  |
| Participants (intercept) | 4.98 |  |  |  |
| Stimulation | 1.75 |  |  |  |
| Response type | 5.01 |  |  |  |
| Stimulation × Response type | 2.18 |  |  |  |
| Residual | 9.60 |  |  |  |

*Note.* The maximal random-effects structure was used in the model. Results are based on 53,093 observations. Statistically significant *p* values (*p* < .05) are shown in bold. OCD = obsessive-compulsive disorder; CI = confidence interval.

*
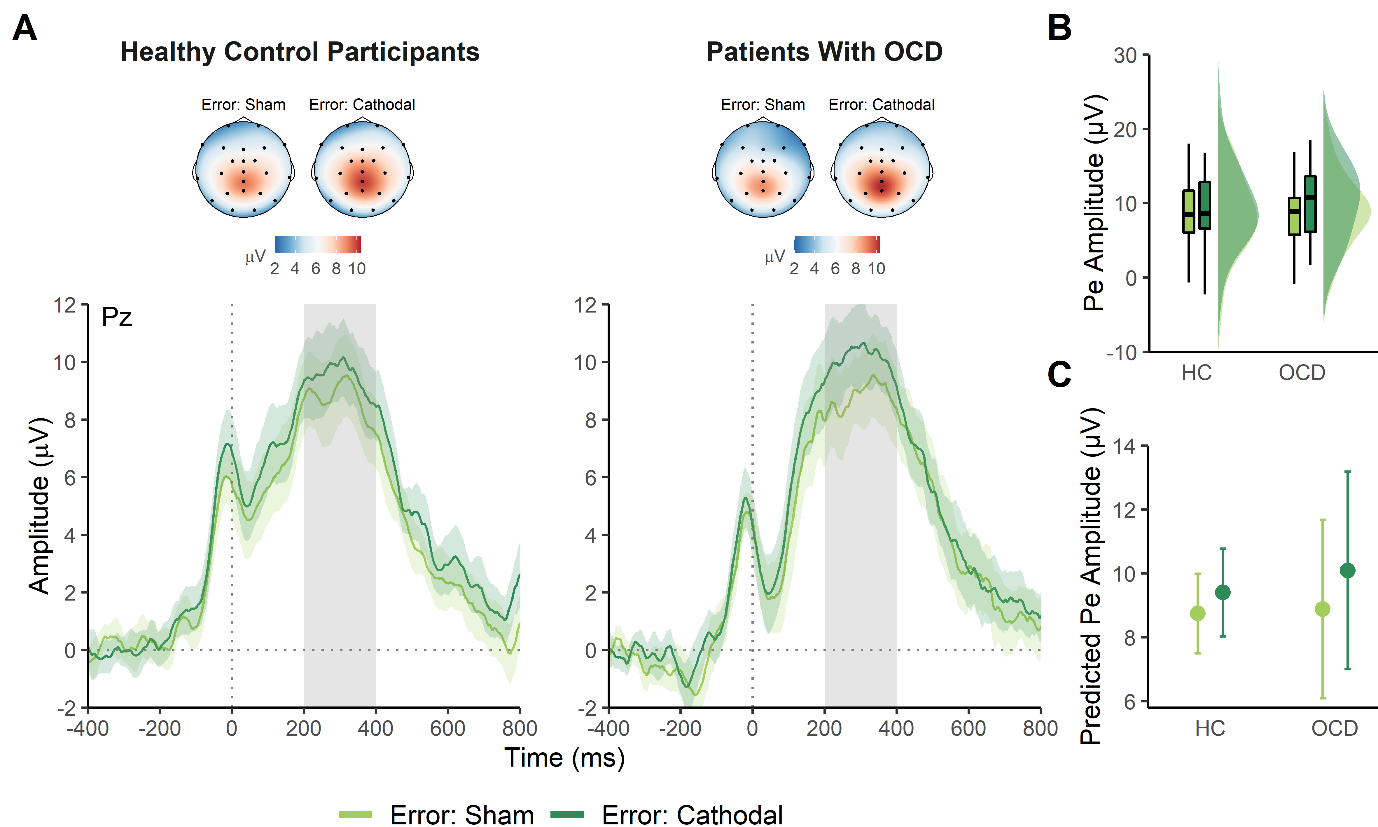
*

***Fig. S1.*** Effects of transcranial direct current stimulation (tDCS) on the error positivity (Pe) in patients with obsessive-compulsive disorder (OCD) and healthy control (HC) participants. **(A)** Response-locked grand average waveforms with 95% confidence intervals (CIs) for incorrect responses at electrode site Pz in the sham and cathodal tDCS condition for patients with OCD and HC participants, along with topographies of the Pe (200–400 ms). Gray-shaded areas in the waveform plots indicate the time window used for Pe quantification. **(B)** Pe mean amplitude values per group and tDCS condition presented as boxplots and probability density plots based on raw data. **(C)** Predicted Pe mean amplitude values per group and tDCS condition calculated as partial effects from a linear mixed model. Error bars represent 95% CIs. **(A–C)** The plots were generated using the packages eegUtils (Version 0.5.0; Craddock, 2020), raincloudplots (Version 0.2.0; Allen et al., 2021), and sjPlot (Version 2.8.6; Lenth, 2020).

**Table S4**

*Results of the Linear Mixed Models (LMMs) Predicting the Error-Related Negativity (ERN), Correct-Response Negativity (CRN), and Error Positivity (Pe) Amplitude as a Function of Stimulation Condition (Cathodal* − *Sham) and Group (OCD_no med_* − *HC; OCD_med_* − *OCD_no med_) to Examine Effects of Psychotropic Medication*

|  | ERN | | | | | |  | CRN | | | |  | Pe | | | |
| --- | --- | --- | --- | --- | --- | --- | --- | --- | --- | --- | --- | --- | --- | --- | --- | --- |
| **Fixed effects** | *b* | | 95% CI | | *t* | *p* | | *b* | 95% CI | *t* | *p* | | *b* | 95% CI | *t* | *p* |
| Intercept | −3.50 | | [−5.29, −1.71] | | −3.84 | **< .001** | | 5.65 | [4.37, 6.93] | 8.66 | **< .001** | | 9.32 | [8.00, 10.65] | 13.80 | **< .001** |
| Stimulation | 0.86 | | [−0.05, 1.77] | | 1.85 | .070 | | 0.83 | [0.21, 1.45] | 2.62 | **.011** | | 0.96 | [0.23, 1.68] | 2.59 | **.012** |
| OCD_no med_ − HC | −1.26 | | [−5.32, 2.79] | | −0.61 | .544 | | −1.67 | [−4.57, 1.23] | −1.13 | .264 | | 1.18 | [−1.82, 4.18] | 0.77 | .446 |
| OCD_med_ − OCD_no med_ | −4.05 | | [−8.85, 0.76] | | −1.65 | .105 | | −1.15 | [−4.58, 2.29] | −0.65 | .516 | | −1.59 | [−5.14, 1.97] | −0.87 | .386 |
| Stimulation × OCD_no med_ − HC | −0.51 | | [−2.56, 1.54] | | −0.49 | .629 | | 0.60 | [−0.81, 2.00] | 0.84 | .407 | | 1.80 | [0.18, 3.43] | 2.17 | **.034** |
| Stimulation × OCD_med_ − OCD_no med_ | | 0.90 | [−1.57, 3.37] | | 0.71 | .480 | | 0.78 | [−0.89, 2.44] | 0.91 | .365 | | −2.66 | [−4.62, −0.70] | −2.66 | **.010** |
| **Random effects** | | *SD* | |  |  |  | | *SD* |  |  |  | | *SD* |  |  |  |
| Participants (intercept) | | 6.31 | |  |  |  | | 4.61 |  |  |  | | 4.64 |  |  |  |
| Stimulation | | 1.76 | |  |  |  | | 2.15 |  |  |  | | 1.32 |  |  |  |
| Residual | | 9.93 | |  |  |  | | 9.58 |  |  |  | | 8.07 |  |  |  |

*Note.* The maximal random-effects structure was used in all models. Results are based on 3,244 and 49,849 observations for ERN/Pe and CRN, respectively. Statistically significant *p* values (*p* < .05) are shown in bold. OCD = obsessive-compulsive disorder; med = medication; HC = healthy control; CI = confidence interval.

**Table S5**

*Results of the Linear Mixed Model (LMM) Predicting the P300 Amplitude as a Function of Stimulation Condition (Cathodal* − *Sham), Group (OCD* − *Healthy Controls), and Response Type (Incorrect* − *Correct)*

| **Fixed effects** | *b* | 95% CI | *t* | *p* |
| --- | --- | --- | --- | --- |
| Intercept | 7.17 | [5.88, 8.45] | 10.90 | **< .001** |
| Stimulation | 0.77 | [0.25, 1.29] | 2.89 | **.006** |
| Group | −1.78 | [−4.35, 0.80] | −1.35 | .182 |
| Response type | −3.52 | [−4.44, −2.61] | −7.52 | **< .001** |
| Stimulation × Group | −0.01 | [−1.05, 1.03] | −0.02 | .980 |
| Stimulation × Response type | 0.28 | [−0.45, 1.02] | 0.76 | .452 |
| Group × Response type | −0.56 | [−2.39, 1.28] | −0.59 | .555 |
| Stimulation × Group × Response type | −0.40 | [−1.87, 1.08] | −0.53 | .601 |
| **Random effects** | *SD* |  |  |  |
| Participants (intercept) | 4.88 |  |  |  |
| Stimulation | 1.63 |  |  |  |
| Response type | 3.30 |  |  |  |
| Stimulation × Response type | 1.63 |  |  |  |
| Residual | 8.16 |  |  |  |

*Note.* The maximal random-effects structure was used in the model. Results are based on 53,002 observations. Statistically significant *p* values (*p* < .05) are shown in bold. OCD = obsessive-compulsive disorder; CI = confidence interval.

**Table S6**

*Results of the Linear Mixed Models (LMMs) Predicting the Error-Related Negativity (ERN), Correct-Response Negativity (CRN), and Error Positivity (Pe) Amplitude as a Function of Stimulation Condition (Cathodal* − *Sham) and Group (OCD* − *Healthy Controls) With Single-Trial P300 Amplitude as a Covariate*

|  | ERN | | | | | |  | CRN | | | |  | Pe | | | |
| --- | --- | --- | --- | --- | --- | --- | --- | --- | --- | --- | --- | --- | --- | --- | --- | --- |
| **Fixed effects** | *b* | | 95% CI | | *t* | *p* | | *b* | 95% CI | *t* | *p* | | *b* | 95% CI | *t* | *p* |
| Intercept | −0.33 | | [−1.75, 1.09] | | −0.46 | .651 | | 5.90 | [4.70, 7.10] | 9.62 | **< .001** | | 11.00 | [9.79, 12.21] | 17.84 | **< .001** |
| Stimulation | 0.91 | | [0.18, 1.64] | | 2.45 | **.034** ^a^ | | 0.67 | [0.09, 1.26] | 2.25 | **.028** | | 0.83 | [0.07, 1.60] | 2.13 | **.038** |
| Group | −2.99 | | [−5.83, −0.14] | | −2.06 | **.044** | | −2.17 | [−4.57, 0.24] | −1.77 | .083 | | 0.65 | [−1.77, 3.07] | 0.53 | .601 |
| P300 | 6.14 | | [5.85, 6.42] | | 42.08 | **< .001** | | 5.65 | [5.59, 5.72] | 161.79 | **< .001** | | 4.09 | [3.84, 4.35] | 31.74 | **< .001** |
| Stimulation × Group | 0.08 | | [−1.38, 1.54] | | 0.11 | .912 | | 0.88 | [−0.30, 2.05] | 1.47 | .148 | | 0.68 | [−0.85, 2.22] | 0.87 | .386 |
| Stimulation × P300 | 0.75 | | [0.20, 1.30] | | 2.66 | **.008** | | 0.09 | [−0.04, 0.23] | 1.34 | .181 | | 0.23 | [−0.26, 0.73] | 0.93 | .354 |
| Group × P300 | −1.01 | | [−1.58, −0.44] | | −3.46 | **.001** | | −0.59 | [−0.73, −0.45] | −8.47 | **< .001** | | −0.34 | [−0.85, 0.16] | −1.34 | .181 |
| Stimulation × Group × P300 | −0.91 | | [−2.01, 0.19] | | −1.62 | .104 | | 0.09 | [−0.19, 0.36] | 0.63 | .528 | | −0.53 | [−1.52, 0.45] | −1.06 | .290 |
| **Random effects** | | *SD* | |  |  |  | | *SD* |  |  |  | | *SD* |  |  |  |
| Participants (intercept) | | 5.29 | |  |  |  | | 4.58 |  |  |  | | 4.49 |  |  |  |
| Stimulation | | 1.50 | |  |  |  | | 2.18 |  |  |  | | 2.04 |  |  |  |
| Residual | | 7.94 | |  |  |  | | 7.72 |  |  |  | | 7.02 |  |  |  |

*Note.* The maximal random-effects structure was used in all models. Results are based on 3,240 and 49,762 observations for ERN/Pe and CRN, respectively. Statistically significant *p* values (*p* < .05) are shown in bold. OCD = obsessive-compulsive disorder; CI = confidence interval.

^a^ Holm–Bonferroni-adjusted *p* value is reported (uncorrected *p* = .017).

**References**

Allen, M., Poggiali, D., Whitaker, K., Marshall, T., van Langen, J., & Kievit, R. (2021). Raincloud plots: A multi-platform tool for robust data visualization. *Wellcome Open Research, 4*(63). <https://doi.org/10.12688/wellcomeopenres.15191.2>

Craddock, M. (2020). *eegUtils: Utilities for electroencephalographic (EEG) analysis* (Version 0.5.0) [Computer software]. <https://doi.org/10.5281/zenodo.3608328>

Lenth, R. (2020). *emmeans: Estimated marginal means, aka least-squares means* (Version 1.5.2-1) [Computer software]. <https://CRAN.R-project.org/package=emmeans>
